# Changes in MiRNA-5196 Expression as a Potential Biomarker of Anti-TNF-α Therapy in Rheumatoid Arthritis and Ankylosing Spondylitis Patients

**DOI:** 10.1007/s00005-018-0513-y

**Published:** 2018-05-09

**Authors:** Marzena Ciechomska, Krzysztof Bonek, Michal Merdas, Patryk Zarecki, Jerzy Swierkot, Piotr Gluszko, Katarzyna Bogunia-Kubik, Wlodzimierz Maslinski

**Affiliations:** 1 Department of Pathophysiology and Immunology, National Institute of Geriatrics Rheumatology and Rehabilitation, Warsaw, Poland; 2 Laboratory of Clinical Immunogenetics and Pharmacogenetics, Hirszfeld Institute of Immunology and Experimental Therapy, Polish Academy of Sciences, Wroclaw, Poland; 3 Department of Rheumatology, National Institute of Geriatrics Rheumatology and Rehabilitation, Warsaw, Poland; 4 Department of Rheumatology and Internal Medicine, Wroclaw Medical University, Wroclaw, Poland; 5 Department of Internal, Occupational Diseases, Hypertension and Clinical Oncology, Wroclaw Medical University, Wroclaw, Poland

**Keywords:** MiRNA, Biomarker, Rheumatoid arthritis, Ankylosing spondylitis, Anti-TNF-α, Biologic therapy

## Abstract

In this study, we analysed the expression level of sera circulating miRNA-5196 in rheumatoid arthritis (RA) and ankylosing spondylitis (AS) patients before and after tumor necrosis factor (TNF)-α therapy as biomarkers predicting positive treatment outcome. We enrolled 10 RA patients, 13 AS patients, and 12 healthy individuals in the study. The expression of miRNA-5196 was measured by real-time polymerase chain reaction before and after anti-TNF-α therapy. Disease activity of RA patients was assessed using disease activity score 28 (DAS28), whereas ankylosing spondylitis DAS (ASDAS) was used in AS patients. MiRNA-5196 expression was significantly higher in patients with RA and AS before TNF-α therapy than in those following anti-TNF-α therapy and healthy controls. Changes in miRNA-5196 expression positively correlated with delta DAS28 or delta ASDAS, respectively, following TNF-α therapy. In contrast, changes in C-reactive protein (CRP) levels in RA and AS patients did not positively correlate with DAS28 or ASDAS changes. Receiver-operating characteristic analysis showed better diagnostic accuracy of miRNA-5196 expression both in RA (area under curve (AUC) = 0.87, *p* = 0.055) and AS patients (AUC = 0.90, *p* = 0.050) compared to CRP levels in RA (AUC = 0.75, *p* = 0.201) and AS patients (AUC = 0.85, *p* = 0.086) upon biologic therapy treatment. Finding novel biomarkers, including miRNA-5196 which allow to predict and monitor anti-TNF-α response, would be of clinical value especially during the early phase of RA or AS development.

## Introduction

Rheumatic diseases, including rheumatoid arthritis (RA), systemic sclerosis (SSc), or ankylosing spondylitis (AS), are chronic autoimmune disorders characterized by pain and joint inflammation (Ciechomska and O’Reilly 2016). In the industrialized world, rheumatic diseases affect more individuals than any other disease group (eular.org https://www.eular.org/myUploadData/files/10%20things%20on%20RD.pdf). In fact, a third of people of all ages are affected by rheumatic diseases at some point during their lifetime (eular.org https://www.eular.org/myUploadData/files/10%20things%20on%20RD.pdf). Rheumatic diseases tend to be progressive in terms of disability; therefore, during the last decade, the economic burden of rheumatic diseases has been increasingly recognised. The cost of rheumatic diseases is estimated at more than 200 billion euros per year in Europe (eular.org https://www.eular.org/myUploadData/files/10%20things%20on%20RD.pdf). In particular, RA affects approximately 1% of Western countries and without optimal treatment approximately 30% of patients with RA become permanently work disabled within 2–3 years of diagnosis (Gibofsky 2012). Unfortunately, there is no cure for rheumatic diseases. Treatment with conventional disease modifying anti-rheumatic drugs (cDMARDs) and/or nonsteroidal anti-inflammatory drugs (NSAIDs) is often associated with various adverse reactions, thus introduction in 1998 of biologic drug therapies provided a new form of treatment (Curtis and Singh 2011). Tumor necrosis factor (TNF)-α plays a pivotal role in tissue destruction and overall RA pathogenesis; therefore, the development of anti-TNF-α therapy has been a milestone in the treatment of RA. Based on experimental and clinical evidences, blockade of TNF-α with antibodies resulted in down-regulation of interleukin (IL)-1, granulocyte–macrophage colony-stimulating factor, IL-6, IL-8 and many other active molecules such as matrix metalloproteinases (MMPs) (Catrina et al. 2002; Maini and Feldmann 2002; Taylor and Feldmann 2009). These factors are involved in joint destruction in RA patients (Brennan et al. 1989). Similarly, patients suffering from AS have shown improvements in pain, functional ability and inflammatory markers such as C reactive protein (CRP) upon anti-TNF-α treatment (Bao et al. 2014; Inman et al. 2008; Wang et al. 2016). In addition, global gene expression studies of AS patients demonstrated that genes involved in Toll-like receptor signaling, TNF signaling, type I interferon signaling and wingless-type MMTV integration site family (Wnt) signaling returned to normal levels following TNF-α inhibitors treatment (Dolcino et al. 2017). These data strongly suggest beneficial role of anti-TNF-α therapy both in RA and AS patients. Unfortunately, the high cost of treatment and the degree of efficacy of anti-TNF-α treatment pose significant problems and fail to provide a solution to all patients (Nair et al. 2016; Schoels et al. 2010). Therefore, there is a strong need to identify the factors which allow to predict a successful outcome before the start of therapy not only in patents suffering for RA but also other rheumatic diseases including AS.

MicroRNAs (miRNAs) are endogenous, non-coding, single-stranded RNAs of approximately 19–25 nucleotides in length. They can negatively regulate gene expression of target messenger RNA (mRNA) (Ciechomska et al. 2014; Macfarlane and Murphy 2010). In addition, miRNAs are attractive as potential biomarkers, since their abnormal expression pattern reflects the underlying pathophysiologic processes (Alevizos and Illei 2010). The expression of miRNAs can be altered under conditions of pathophysiological stress, disease or treatment (Mendell and Olson 2012). Thus, identification of miRNAs which will be effective as biomarkers determining early diagnosis and response to anti-TNF-α therapy might be of great interest in RA and AS. In this study, we evaluated whether changes in miRNA-5196 expression can be used as a biomarker predicting the positive outcome of anti-TNF-α therapy in RA and AS patients.

## Materials and Methods

### Sample Collection and Cell Purification

Ten patients who fulfilled the American College of Rheumatology (ACR) and the European League Against Rheumatism (EULAR) criteria (Aletaha et al. 2010) for the classification of RA were obtained from Wroclaw Medical University. Their clinical characteristics are summarized in Table 1. Thirteen patients who fulfilled the classification of ASAS criteria from 2009 for the diagnosis of AS (Rudwaleit et al. 2009) were obtained from National Institute of Geriatrics Rheumatology and Rehabilitation in Warsaw. Their clinical characteristics are summarized in Table 2. These studies were approved by the local ethics committees (approval no. 335/2014 and KBT-1/6/2017) and all RA and AS patients provided fully informed written consent. Fifteen healthy donors with no history of autoimmune disease were included as healthy control (HC). The blood from HC was collected from a local blood donor centre or directly from healthy volunteers. The serum samples from HC, RA and AS patients were collected in serum separation tubes (BD Vacutainer® SST II Plus), aliquoted and frozen at − 80 °C. To determine correct disease activity score 28 (DAS28) in RA patients, the level of CRP was used in formula. Similarly, CRP was used as one of the parameters to estimate the ASDAS score.

**Table 1:** Clinical and laboratory data of RA patients

Parameters of RA patients (*n* = 10)	RA patients
Age, years, median (range)	59 (27–74)
Sex F/M	6/4
Disease duration, years, median (range)	7 (2–25)
Anti-CCP Abs % (*n*)	30% (*n* = 3)
RF % (*n*)	90% (*n* = 9)
CRP, mg/L, median (range) before anti-TNF-α	10.15 (1.9–30.2)
CRP, mg/L, median (range) after anti-TNF-α	3.4 (3.4–27.9)
BMI median (range)	24 (18–25)
Smoker, % (*n*)	0% (*n* = 0)
Treatment	
Etanercept, % *(n)*	70% (*n* = 7)
Adalimumab, % *(n)*	30% (*n* = 3)
Methotrexate, % (dose of treated patients)	100% (10–25 mg)
Average DAS28 before treatment (range)	6.77 (6.16–7.77)
Average DAS28 after treatment (range)	4.48 (3.69–5.31)

**Table 2:** Clinical and laboratory data of AS patients

Parameters of AS patients (*n* = 13)	AS patients
Age, years, median (range)	50 (32–59)
Sex F/M	10/3
Disease duration, years, median (range)	9.5 (1–37)
HLA-B27% (*n*)	77% (*n* = 10)
Iritis (*n*)	31% (*n* = 4)
CRP, mg/L, median (range) before anti-TNF-α	7 (3–61)
CRP, mg/L, median (range) after anti-TNF-α	6 (2–15)
BMI median (range)	23 (18–25)
Smoker, % (*n*)	23% (*n* = 3)
Treatment	
Golimumab, % *(n)*	15% (*n* = 2)
Adalimumab, % *(n)*	77% (*n* = 10)
Certolizumab, % (*n*)	8% (*n* = 1)
Average ASDAS before treatment (range)	3.64 (1.8–4.8)
Average ASDAS after treatment (range)	2.29 (1–3.5)
Average BASDAI before treatment (range)	6.10 (2.8–8.8)
Average BASDAI after treatment (range)s	3.48 (1–8)
Average HAQ before treatment (range)	1.19 (0.37–3.75)
Average HAQ after treatment (range)	0.57 (0–2.12)

### Gene Expression Study

The volume of 300 µl of sera from HC, RA and AS patients was used to isolate circulating miRNA-5196 using NucleoSpin® miRNA plasma/serum (Macherey–Nagel, Germany) according to the manufacturer’s protocol. TaqMan ® microRNA RT Kit (Thermo Fisher Scientific, USA) was used to reverse transcribe to cDNA with the use of TaqMan® MicroRNA Assays for hsa-miR-5196-5q (471527_mat), and hsa-let-7a (000377) all from Thermo Fisher Scientific (USA). Based on previously published papers, hsa-let-7a was used as an internal control to normalize miRNA-5196 input (Davoren et al. 2008; Li et al. 2015). The 20 µL PCR reaction included 1.33 µL RT product, 1× TaqMan Universal PCR master mix and 1 µL primers and probe mix of the TaqMan MicroRNA Assay Kit (Thermo Fisher Scientific, USA). Reactions were performed at 95 °C for 10 min, followed by 50 cycles at 95 °C for 15 s and 60 °C for 1 min. Samples were analysed in triplicate using the Viia 7 or QuantStudio 5 qRT-PCR machines (Thermo Fisher Scientific, USA). The expression levels relative to the average HC (arbitrarily set at 1) were calculated using the following equation: (2^Delta Delta CT) − 1, all normalized to hsa-let-7a internal control.

### Statistical Analysis

All data are presented as mean ± SEM. D’Agostino & Pearson normality test was used to confirm the use of parametric or non-parametric test for the further analysis. The differences between the groups were tested for their statistical significance using either *t* test or Mann–Whitney *U* test. A *p* value of less than 0.05 was considered statistically significant; *p* values are expressed as follows: ns for not significant; 0.05 > *p* > 0.01 as *; 0.01 > *p* > 0.001 as **; *p* < 0.001 as ***. Correlations between delta DAS28 or ASDAS and delta miRNA-5196 and delta CRP following TNF-α therapy in RA or AS patients, respectively, were analysed using the non-parametric correlation Spearman analysis. To validate the accuracy of biomarkers, receiver-operating characteristic (ROC) curve analysis was introduced. GraphPad Prism (GraphPad Software) was used to calculate all tests. The cut-off value was established based on the Youden Index which allows to demonstrate the maximum potential effectiveness of a biomarker.

## Results

### The Expression of Sera Circulating MiRNA-5196 is Elevated in Patients with Autoimmune Rheumatic Diseases Including SSc, RA and AS Compared to HC

**Fig. 1:**
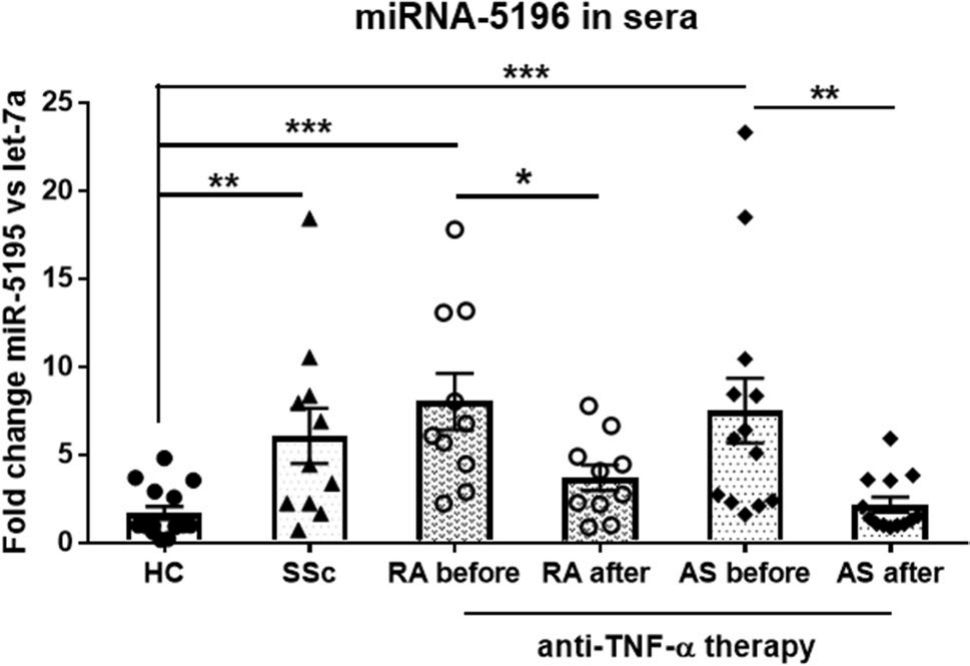
Level of circulating miRNA-5196 present in sera of rheumatic disease patients. The expression level of sera circulating miRNA-5196 was measured in HC (*n* = 15), SSc (*n* = 11) patients and in RA patients before (*n* = 10) and after (*n* = 10) TNF-a therapy and in AS patients before (*n* = 13) and after (*n* = 13) TNF-a therapy. Results were normalized to the let-7a internal control. Each symbol represents an individual subject; horizontal lines with bars show the mean ± SEM. *P* values are expressed as follows: 0.05 > *P* > 0.01 as *; 0.01 > *P* > 0.001 as **; *P* < 0.001 as ***

Since we have previously demonstrated that the level of miRNA-5196 is increased in sera and monocytes isolated from SSc patients compared to HC (Ciechomska et al. 2017), we sought to investigate the level of miRNA-5196 in sera from RA and AS patients to determine whether circulating miRNA-5196 can be used as a potential biomarker of rheumatic diseases. As a positive control of increased miRNA-5196 expression we used SSc sera. Indeed, the expression of SSc miRNA-5196 was significantly enhanced (3.57-fold, *p* = 0.006) compared to HC sera. The level of circulating miRNA-5196 was also significantly elevated in other rheumatic diseases. In RA and AS patients, the level of miRNA-5196 was 4.72-fold (*p* = 0.0001) and 4.41-fold (*p* = 0.0003), increased, respectively compared to HC sera (Fig. 1). Of note, in those RA patients who were undergoing 6 months anti-TNF-α therapy, the level of circulating miRNA-5196 was decreased but still significantly higher than HC (2.18-fold, *p* = 0.012,). In AS patients treated with TNF-α inhibitors the expression level of miRNA-5196 was also reduced and reached almost HC sera level (1.27-fold, *p* = 0.117). This suggests that expression of circulating miRNA-5196 from patients suffering from rheumatic diseases including SSc, RA and AS is significantly elevated and it can be reduced by anti-TNF-α treatment.

### Treatment with Biologic Therapy is Accompanied with Significant Down-Regulation of MiRNA-5196 Expression but not with Reduction of CRP Level

**Fig. 2:**
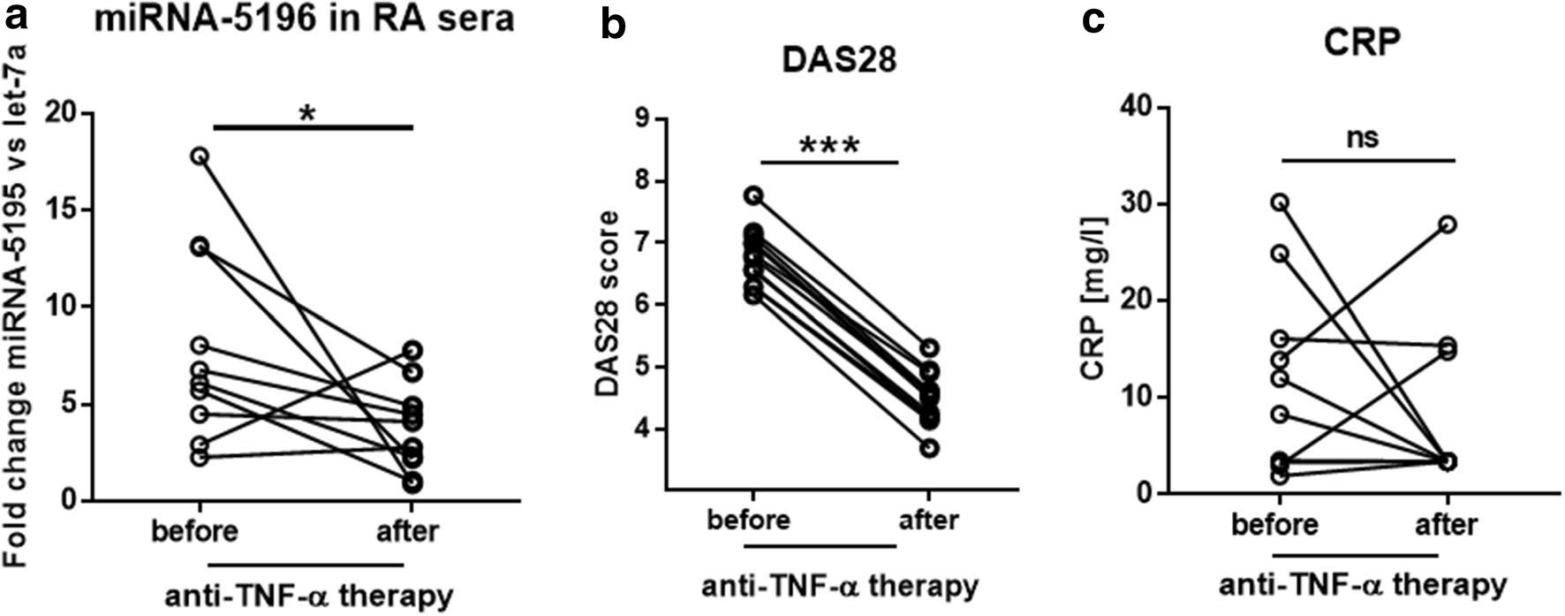
Expression level of miRNA-5196, DAS28 score and CRP before and after anti-TNF-a therapy. The expression level of sera circulating miRNA-5196 (**a**), disease activity score DAS28 (**b**) and CRP (**c**) were measured in RA patients before (*n* = 10) and after (*n* = 10) TNF-a therapy. *P* values are expressed as follows: 005 > *P* > 001 as *; 001 > P > 0001 as **; *P* < 0001 as ***, *ns* not significant

**Fig. 3:**
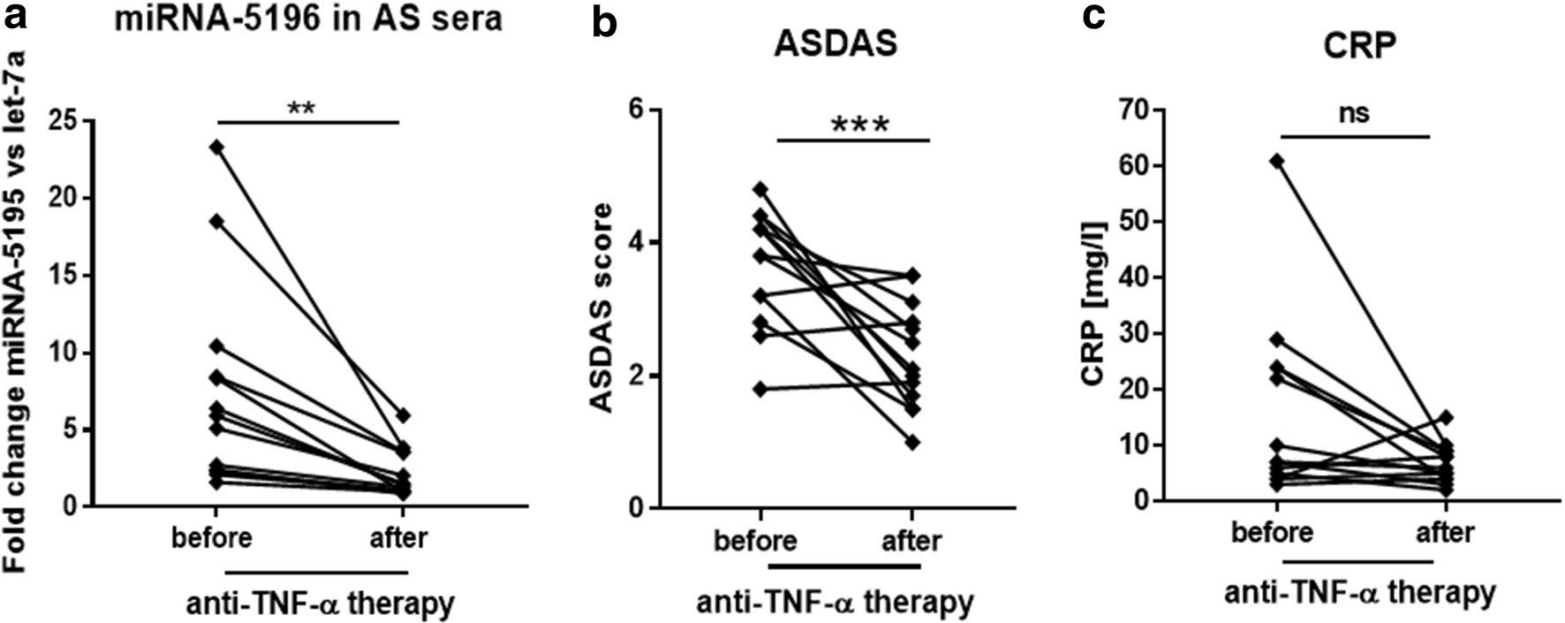
Expression level of miRNA-5196, DAS28 score and CRP before and after anti-TNF-a therapy. The expression level of sera circulating miRNA-5196 (**a**), ankylosing spondylitis disease activity score ASDAS (**b**) and CRP (**c**) were measured in AS patients before (*n* = 13) and after (*n* = 13) TNF-a therapy. *P* values are expressed as follows: 005 > *P* > 001 as *; 001 > *P* > 0001 as **; *P* < 0001 as ***, *us* not significant

Apart from two individuals, the expression of miRNA-5196 was significantly decreased (2.16-fold, *p* = 0.024) in all RA patients following anti-TNF-α treatment (Fig. 2a). Simultaneously, the average DAS28 score was also significantly reduced (*p* < 0.0001) from 6.77 to 4.48 in RA patients who were undergoing 6 months biologic therapy (Fig. 2b) whereas inflammation parameter including CRP did not drop significantly (*p* = 0.67) (Fig. 2c). Regarding AS patients we have also noticed significant reduced expression of miRNA-5196 (3.45-fold, *p* = 0.009) (Fig. 3a) and ASDAS (1.59-fold, *p* = 0.004) (Fig. 3b) following biologic agent treatment. Similarly to RA patients, the CRP level in AS patients did not drop significantly (*p* = 0.20) (Fig. 3c). Thus, we speculate that reduction in miRNA-5196 expression, but not decreased level of CRP, might a good marker of anti-TNF-α therapy response in RA and AS patients.

### Changes in MiRNA-5196 Expression Can be Used as a Good Marker for Predicting Reduced Disease Activity Score upon TNF-α Inhibitors Treatment in RA and AS Patients

**Fig. 4:**
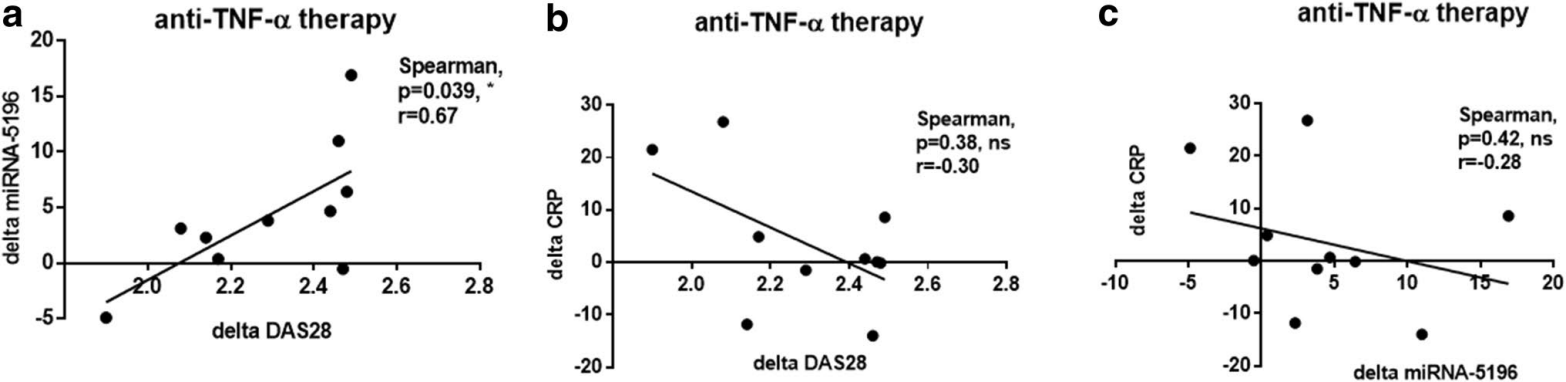
Correlation between changes in serum level of miRNA-5196 (delta miRNA-5196), changes in DAS28 (delta DAS28) and changes in CRP levels (delta CRP) in RA patients (*n* = 10) following anti-TNF-a therapy. Changes in miRNA-5196 expression were correlated with changes in DAS28 (**a**), changes in CRP levels were correlated with changes in DAS28 (**b**) and changes in CRP levels were correlated with changes in miRNA-5196 expression (**c**)

**Fig. 5:**
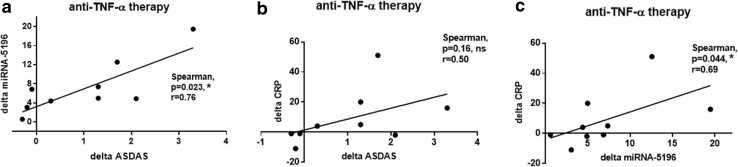
Correlation between changes in serum level of miRNA-5196 (delta miRNA-5196), changes in ASDAS (delta ASDAS) and changes in CRP levels in AS patients (*n* = 9) following anti-TNF-a therapy. Changes in miRNA-5196 expression were correlated with changes in ASDAS (**a**), changes in CRP levels were correlated with changes in ASDAS (**b**) and changes in CRP levels were correlated with changes in miRNA-5196 expression (**c**)

In addition, we have also calculated delta miRNA-5196 expression and delta CRP based on values just before and after anti-TNF-α treatment. Subsequently, these results were correlated with delta DAS28 to compare which parameter, either delta miRNA-5196 or delta CRP, will yield better outcome of anti-TNF-α therapy in RA and AS patients. Interestingly, we noticed that delta miRNA-5195 expression positively correlates with delta DAS28 (*p* = 0.039, *r* = 0.67) (Fig. 4a), whereas changes in CRP level did not correlate with delta DAS28 in RA patients (*p* = 0.38, *r* = − 0.30) (Fig. 4b). Of note, we observed that delta CRP revealed even a negative trend with delta DAS28 (*p* = 0.38, *r* = − 0.30) (Fig. 4b) and delta miRNA-5196 (*p* = 0.42, *r* = − 0.28) (Fig. 4c). In AS patients, delta miRNA-5196 expression was also positively correlated (*p* = 0.02, *r* = 0.75) with delta ASDAS (Fig. 5a) and delta CRP (*p* = 0.04, *r* = 0.69) (Fig. 5c). As previously shown for RA, delta CRP did not correlate with delta ASDAS (*p* = 0.16, *r* = 0.50) (Fig. 5b). These results strongly suggest that changes in miRNA-5196 expression serve as a better predictive biomarker of anti-TNF-α response than changes in CRP level both in RA and AS patients. Due to a small change in miRNA-5196 expression before and after biologic therapy, we have excluded four patients’ samples from the analysis. In Fig. 5, we eliminated the delta miRNA-5196 samples which were within the range between 1 and 1.5. In RA patients, delta miRNA-5196 was substantial and always out of this range; therefore, all patients were included in the correlation analysis (Fig. 4).

### Delta MiRNA-5196 is a More Accurate Biomarker to Monitor Changes of Clinical Activity of AS and RA Patients than Delta CRP

**Fig. 6:**
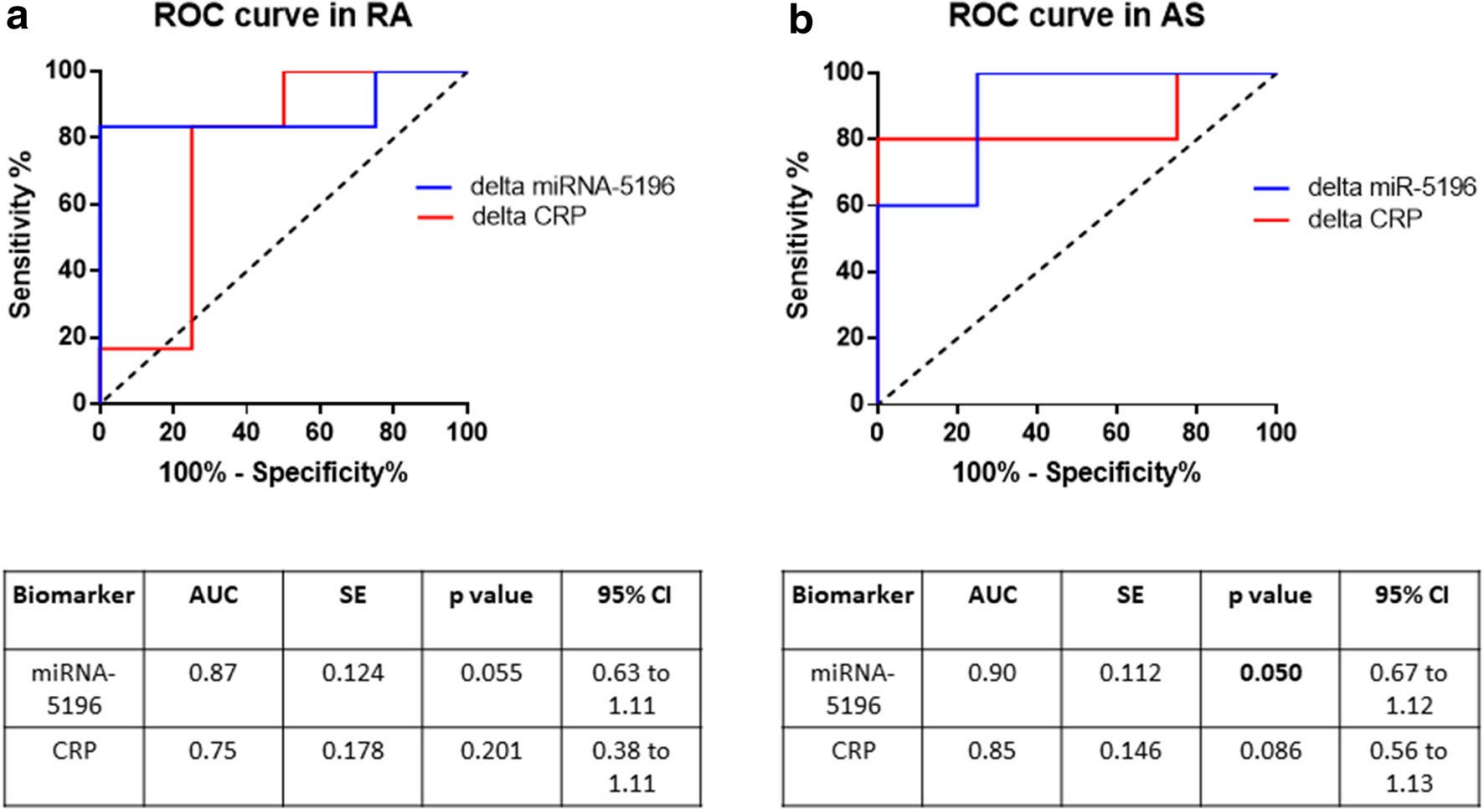
ROC analysis comparing delta miRNA-5196 and delta CRP levels as biomarkers predicting anti-TNF-a outcomes in RA (**a**) and AS patients (**b**). 10 RA patients and 9 AS patients were included in the analysis

Finally, to evaluate the diagnostic potential of circulating miRNA-5196 as a useful detection biomarker predicting response to a biologic therapy, ROC curve analysis was performed. In Fig. 6a, b, it can be seen that delta miRNA-5196 was much more specific in predicting anti-TNF-α response (area under curve (AUC) = 0.87, CI = 0.63–1.11, *p* = 0.055 and AUC = 0.90, CI = 0.67–1.21, *p* = 0.050, respectively, in RA and AS patients) than delta CRP level (AUC = 0.83, CI = 0.56–1.10, *p* = 0.088 and AUC = 0.85, CI = 0.56–1.13, *p* = 0.086, respectively, in RA and AS patients). These results indicate that sera miRNA-5196 can be a better biomarker to measure reduced disease activity upon biologic therapy treatment than CRP level both in RA and AS patients. Based on the Youden Index, at a cut-off value of 3.81 for delta miRNA-5196, sensitivity and specificity values were 83 and 100%, respectively, for RA patients, whereas at a cut-off value of 0.7 for delta CRP, sensitivity and specificity values were 83 and 75%, respectively. In AS patients, at a cut-off value of 4.89 for delta miRNA-5196, sensitivity and specificity values were 100 and 75%, respectively, for RA, whereas at a cut-off value of 5 for delta CRP, sensitivity and specificity values were 80 and 100%, respectively.

## Discussion

In this study, we investigated whether sera circulating miRNA-5196 can be used as a versatile biomarker characterising autoimmune rheumatic diseases including RA, SSc and AS. In addition, we examined whether changes in miRNA-5196 expression can be used as a better predictor for clinical response to anti-TNF-α therapy than changes in CRP levels in RA and AS patients.

RA is a complex autoimmune disease characterized by inflammation and joint destruction. Similarly, AS is characterized by joint inflammation that primarily affects the spine. Overall, both diseases result in stiffness, pain and joint deformities. Although, the management of rheumatic diseases has undergone major advances in recent years, both in terms of the drugs discovery and therapeutic strategy but still pose a significant socioeconomic problem (Klak et al. 2016). Unfortunately, current treatment still does not guarantee complete recovery of patients with rheumatic diseases and it is only possible to slow down or reduce the disease activity. Thus, the development of more effective diagnosis of rheumatic diseases are badly needed to increase patients’ functioning and quality of life. Inflammation in RA and AS is mediated mostly by TNF-α; therefore, the introduction of biologic therapy targeting this cytokine has revolutionised RA and AS treatment. Unfortunately, high costs of anti-TNF-α treatment requires good predictors to reduce the economic burden of RA treatment. Indeed, there are currently no straightforward predictors of a good response to anti-TNF-α drugs and significant percentage of patients fail to respond to treatment (25–38% of etanercept patients, 21–42% of infliximab patients) (Seymour et al. 2001). Recent studies have shown that miRNA play an important role in RA and AS diagnosis and dysregulated miRNA expression seems to contribute to the molecular mechanisms of the disease (Chen et al. 2015; Li et al. 2016; Prajzlerova et al. 2017). MiRNAs are short (approximately 21 nucleotides long) non-coding RNAs that can influence mRNA processing at the post-transcriptional level. It has been shown that plasma miRNA signature of miRNA-23 and miRNA-223 was used as predictor and biomarker of response to anti-TNF-α/DMARDs combination therapy in RA patients (Castro-Villegas et al. 2015). Indeed, upregulated expression of miRNA-23 and miRNA-223 was inversely correlated with secretion of TNF-α, IL-6, IL-17, rheumatoid factor and CRP in responder patients (Castro-Villegas et al. 2015). Expression of miRNA-451 in T cells also positively correlated with DAS28 (Smigielska-Czepiel et al. 2014). Another study has shown that expression of miRNA-125b in peripheral blood mononuclear cells was a better predictor for an optimal treatment outcome after 3 months TNF-α therapy than baseline levels of DAS28, CRP, erythrocyte sedimentation rate (ESR) (Hruskova et al. 2016). In the recent studies, it has been observed that sera circulating miRNA-29a-3p, miRNA-146a-5p or miRNA-222-3p were associated with spinal changes and/or disease activity assessed by bath ankylosing spondylitis disease activity index (BASDAI) in AS patients (Prajzlerova et al. 2017). Interestingly, AS patients receiving anti-TNF-α therapy exhibited significantly lower levels of selected miRNAs than anti-TNF-α naive patients (Prajzlerova et al. 2017). Previously, we have also demonstrated that miRNA-135b targeting STAT3 is reduced in other rheumatic diseases including SSc (O’Reilly et al. 2016). Whereas, miRNA-5196 is elevated in SSc monocytes and SSc sera, suggesting that these miRNAs can be used as potential biomarkers characterising SSc. In addition, high-throughput Solexa deep sequencing analysis followed by computational analysis identifies miRNA-5196 as one of novel candidates characterising different types of childhood acute lymphoblastic leukemia (ALL) (Schotte et al. 2011). Indeed, miRNA-5196 has been listed as a top 10 of novel miRNAs with highest read frequency in ALL patients. Similarly, miRNA-5196 was upregulated in esophageal cancer (Liao et al. 2016). In contrast, the expression of miRNA-5196 in serum of colon cancer patients was downregulated compared to HC suggesting that circulating miRNA-5196 may be applied in early tumor diagnosis, prognosis and recurrence with a great value (Liu et al. 2015; Zhang et al. 2017). Based on prediction algorithms obtained from miRDB, Target Scan Human and miRanda databases, miRNA-5196 has been described as a negative regulator of Fra2 (AP-1 family transcription factor) and MMP-15, IL-1 receptor type I. These molecules play an important role in inflammation and progression of rheumatic diseases (Araki and Mimura 2017; Dey et al. 2016; Magyari et al. 2014). Indeed, in the previous paper we have shown that miRNA-5196 targets Fra2 and subsequently reduces profibrotic TIMP-1 production in SSc monocytes (Ciechomska et al. 2017). MiRNA-5196 binds to five seed regions within 3′UTR of Fra2, therefore, can negatively regulate gene expression of target mRNA (Ciechomska et al. 2017). Thus, we sought to investigate whether miRNA-5196 could be used as a novel biomarker for predicting anti-TNF-α therapy in RA and AS patients. Indeed, in the present study we have shown that sera circulating miRNA-5196 is elevated in SSc, RA and AS patients compared to HC (Fig. 1). Following anti-TNF-α therapy, the level of miRNA-5196 was reduced in RA patients and in AS patients. Reduced expression of miRNA-5196 was seen in 80% of RA patients responding to anti-TNF-α therapy and in all AS patients (Fig. 2). Similar to the previously published results (Ciechomska et al. 2017), we have also seen increased expression of miRNA-5196 in SSc sera (Fig. 1). In addition, we have demonstrated that a change in miRNA-5196 expression positively correlates with changes in clinical parameters (DAS28 and ASDAS) upon biologic therapy treatment in RA and AS patients, respectively (Figs. 4a, 5a). In contrast, in Figs. 4b and 5b, it can be seen that alteration in the CRP levels did not associate with clinical parameters in RA and AS patients. These results suggests that changes in miRNA-5196 expression occurred during TNF-α therapy could support more informed clinical decisions on the most appropriate treatment regimens for individual RA and AS patients than changes in CRP levels. Although changes in the CRP levels are considered as a gold standard to evaluate therapy in rheumatic diseases, some studies have shown controversial role of CRP level as a biomarker for disease activity. In systemic lupus erythematosus patients there were no correlations between CRP serum levels or anti-CRP antibodies and disease activity (Rezaieyazdi et al. 2011; Son et al. 2017), suggesting that CRP may not be an ideal indicator for disease activity. Furthermore, using ROC analysis evaluating the diagnostic accuracy of biomarkers, we have observed that delta miRNA-5196 was a better predictor for a clinical response to personalised biologic therapy than delta CRP in RA and AS patients. Indeed, we have observed larger AUC values for delta miRNA-5196 (0.87 for RA and 0.90 for AS) compared to AUC values for delta CRP (0.75 for RA and 0.85 for AS).

Taken together, we have shown that changes in miRNA-5196 expression holds promise as a good marker of anti-TNF-α treatment response. A clear limitation of our study is a relatively small sample size of the groups. Ideally, validation of miRNA-5196 expression as a prognostic marker in large cohorts of patients may allow for better selection of RA and AS patients benefiting the most from anti-TNF-α therapy. Therefore, more studies exploring the value of miRNA-5196 as a predictive biomarker for treatment response would be of interest and may facilitate their introduction into clinical practice.
